# Supplementary material for: The OWL Screening Tool—A Protocol for Holistic Pediatric Lifestyle Assessment
Source: Healthcare (Basel). 2025 Oct 28;13(21):2731. doi: 10.3390/healthcare13212731 (PMC12609874; doi:10.3390/healthcare13212731)
Supplement: Supplementary file 1 [file healthcare-13-02731-s001.zip › healthcare-3942866-supplementary.pdf]

Supplementary File S1. The template provides an example of screening school children aged 6 to 12 years in Arabic language

لا	نعم	سؤال
		**أ. التغذية**
		هل تشرب حوالي 1.2 لتر من الماء (حوالي 40 مل / كجم من وزن الجسم) كل يوم؟
		هل تتناول الحبوب الكاملة كل يوم (دقيق الشوفان، الخبز والأرز الكامل، الكينوا، الشعير، المعكرونة الكاملة، الخ)؟
		هل تتناولين على الأقل حصتين من الفاكهة و2 إلى 3 حصص من الخضروات الطازجة أو المطبوخة يوميًا؟*
		هل تتناول 3 وجبات يوميًا تحتوي على جزء من الأطعمة الغنية بالبروتين (منتجات الألبان والبيض والأسماك واللحوم والبقوليات والتوفو والمكسرات والبذور)؟*
		هل تتناول المكسرات بانتظام (2-3 مرات على الأقل في الأسبوع)؟
		**ب. النشاط البدني**
		هل تصل إلى ١٠٠٠٠ خطوة يوميًا؟ (دقيقة تقريبًا - ١٠٠ خطوة، ١٠٠ دقيقة - ١٠٠٠٠ خطوة)
		هل تقضي في المتوسط 60 دقيقة يوميًا في ممارسة نشاط بدني متوسط إلى مكثف (الجري، السباحة، ركوب الدراجات، الرياضة المدرسية)؟**
		هل تقوم بتدريب قوة العضلات ثلاث مرات في الأسبوع على الأقل (التمرين في المدرسة، الركض، تدريب الأثقال)؟
		هل تلعب ألعابًا مثل كرة القدم أو كرة السلة أو الرياضات التنسيقية أو الرقص أو فنون الدفاع عن النفس أو اليوجا وما إلى ذلك مرة واحدة على الأقل في الأسبوع؟
		هل تمارس نشاطًا بدنيًا كل يوم في الهواء الطلق (الرياضة المدرسية، ركوب الدراجات، السباحة، المشي، ممارسة الألعاب النشطة)؟
		**ج. النوم**
		هل تنام من 9 إلى 11 ساعة كل ليلة؟
		هل تنام جيدا في الليل؟
		هل تذهب إلى السرير ما بين الساعة 9 و 10 مساءً على الأقل؟
		هل لديك الوقت الكافي لتناول وجبة الإفطار بعد الاستيقاظ، قبل المدرسة؟
		هل تمارس طقوس المساء (المشي، التنفس، الصلاة، الامتنان، الصمت، الخ) قبل ساعة على الأقل من الذهاب إلى السرير؟
		**د. إدارة الإجهاد**
		هل تستمتع بهواية كل أسبوع (الرياضة، الفن، البستنة، الطبخ، الخياطة، الغناء، القراءة وما إلى ذلك)؟
		هل تلتقي بأصدقائك لقضاء وقت ممتع كل أسبوع؟
		هل تمارس تقنيات الاسترخاء كل يوم (التنفس، واليوغا، والتأمل، وما إلى ذلك)؟
		هل تمارس الأسرة أنشطة ترفيهية معًا بشكل منتظم (على الأقل في عطلات نهاية الأسبوع والأعياد الرسمية)؟
		هل وضعت عائلتك قواعد محددة لوقت الشاشة؟
		**معايير الحصول على نقطة واحدة لكل إجابة إيجابية بنعم على السؤال**
